# Improvements in the South African HIV care cascade: findings on 90‐90‐90 targets from successive population‐representative surveys in North West Province

**DOI:** 10.1002/jia2.25295

**Published:** 2019-06-12

**Authors:** Sheri A Lippman, Alison M El Ayadi, Jessica S Grignon, Adrian Puren, Teri Liegler, W D Francois Venter, Mary J Ratlhagana, Jessica L Morris, Evasen Naidoo, Emily Agnew, Scott Barnhart, Starley B Shade

**Affiliations:** ^1^ Center for AIDS Prevention Studies Department of Medicine University of California San Francisco CA USA; ^2^ Bixby Center for Global Reproductive Health Department of Obstetrics, Gynecology and Reproductive Sciences University of California San Francisco CA USA; ^3^ Department of Global Health University of Washington Seattle WA USA; ^4^ International Training and Education Center for Health (I‐TECH) South Africa Pretoria Republic of South Africa; ^5^ Centre for HIV and STIs National Institute for Communicable Diseases/NHLS Division of Virology School of Pathology University of the Witwatersrand Johannesburg South Africa; ^6^ HIV/AIDS Division Department of Medicine HIV, Infectious Diseases and Global Health Division University of California San Francisco CA USA; ^7^ Wits Reproductive Health and HIV Institute (WRHI) Faculty of Health Sciences University of the Witwatersrand Johannesburg South Africa; ^8^ Institute for Global Health Science Department of Epidemiology and Biostatistics University of California San Francisco CA USA

**Keywords:** South Africa, HIV, care cascade, 90‐90‐90 targets, HIV care continuum, linkage to care, viral suppression, testing

## Abstract

**Introduction:**

To achieve epidemic control of HIV by 2030, countries aim to meet 90‐90‐90 targets to increase knowledge of HIV‐positive status, initiation of antiretroviral therapy (ART) and viral suppression by 2020. We assessed the progress towards these targets from 2014 to 2016 in South Africa as expanded treatment policies were introduced using population‐representative surveys.

**Methods:**

Data were collected in January to March 2014 and August to November 2016 in Dr. Ruth Segomotsi Mompati District, North West Province. Each multi‐stage cluster sample included 46 enumeration areas (EA), a target of 36 dwelling units (DU) per EA, and a single resident aged 18 to 49 per DU. Data collection included behavioural surveys, rapid HIV antibody testing and dried blood spot collection. We used weighted general linear regression to evaluate differences in the HIV care continuum over time.

**Results:**

Overall, 1044 and 971 participants enrolled in 2014 and 2016 respectively with approximately 77% undergoing HIV testing. Despite increases in reported testing, known status among people living with HIV (PLHIV) remained similar at 68.7% (95% Confidence Interval (CI) = 60.9–75.6) in 2014 and 72.8% (95% CI = 63.6–80.4) in 2016. Men were consistently less likely than women to know their status. Among those with known status, PLHIV on ART increased significantly from 80.9% (95% CI = 71.9–87.4) to 91.5% (95% CI = 84.4–95.5). Viral suppression (<5000 copies/mL using DBS) among those on ART increased significantly from 55.0% (95% CI = 39.6–70.4) in 2014 to 81.4% (95% CI = 72.0–90.8) in 2016. Among all PLHIV an estimated 72.0% (95% CI = 63.8–80.1) of women and 45.8% (95% CI = 27.0–64.7) of men achieved viral suppression by 2016.

**Conclusions:**

Over a period during which fixed‐dose combination was introduced, ART eligibility expanded, and efforts to streamline treatment were implemented, major improvements in the second and third 90‐90‐90 targets were achieved. Achieving the first 90 target will require targeted and improved testing models for men.

## 1 Introduction

The global HIV response has gained considerable momentum in increasing testing and treatment availability in the most heavily burdened regions. Spurred by findings that viral suppression would benefit not only people living with HIV (PLHIV) but also prevent transmission to uninfected sexual partners 1, international agencies and governments have instituted policies and targets to rapidly expand treatment access. UNAIDS first introduced testing and treatment targets in 2014 which specify that by 2020, 90% of HIV‐positive people are aware of their status, 90% of those diagnosed receive sustained antiretroviral therapy (ART) and 90% of those on ART achieve viral suppression 2. Governments adopted these targets and have more than doubled the number of people on ART in recent years. For example, the proportion of PLHIV on ART in Eastern and Southern Africa increased from 24% in 2010 to 54% in 2015 3, and reached 60% by 2017 4.

Home to the most PLHIV, with an estimated HIV prevalence of 20.6% among adults 15 to 49, 5 and the largest national treatment programme, South Africa has progressively expanded eligibility criteria for ART access 6 and ART availability in primary health settings 7. In 2012, 37.8% of HIV‐positive men and 55.0% of HIV‐positive women were aware of their HIV status 8, 63.1% of men and 68.4% of women in care were on ART, and 73.7% of those on treatment achieved viral suppression 9. ART initiation criteria were extended from CD4 <200 to CD4 <350 in 2013, then from CD4 <350 to CD4 <500 in January 2015, with universal test and treatment (ART initiation regardless of CD4 count) introduced in September 2016 10. In addition, the simplicity of the first‐line regimen improved, with South Africa rolling‐out fixed dose combination (FDC) first‐line ART (comprised of Tenofovir/Emtricitabine/Efavirenz) in April 2013 11. Finally, additional efforts to streamline treatment with strategies such as providing fast track ART pick‐up with longer medication supplies for stable patients were introduced in 2016 12. Introduction of FDC, expanded ART eligibility, and new measures to streamline treatment and improve adherence are likely to make a large impact on care engagement and adherence, moving South Africa closer to meeting the 90‐90‐90 targets following these policy changes. In fact, the summary findings of the National HIV Prevalence, Incidence, Behaviour and Communication Survey 2017 note recent improvements in 90‐90‐90 targets. Although targets vary regionally, survey findings estimated that nationally, 78.0% and 88.9% of HIV‐positive males and females respectively were aware of their status; 67.4% and 72.2% of males and females aware of their status were on ART; and 82.1% and 89.9% and of males and females on ART were virally suppressed 5.

We implemented two sequential population‐representative surveys in 2014 and 2016 in two municipalities in North West Province (NW). The surveys were conducted in conjunction with an HIV prevention programme, providing an opportunity to both evaluate the impact of an HIV prevention programme focusing on increasing HIV testing uptake through community‐based testing campaigns and home‐based testing services, and to assess trends in the HIV care continuum in an understudied but high prevalence area. While we had hypothesized that HIV testing and care engagement would increase in the municipality receiving the combination prevention programme compared to the municipality not receiving these services, we found that changes in the 90‐90‐90 indicators between the two communities were equivalent. However, changes over time in the 90‐90‐90 indicators in both communities reflect trends with important implications in evaluating the impact of national policies because the surveys were implemented at different stages of national ART access expansion. As a result, we describe changes in the 90‐90‐90 targets through comparison of population‐based data from the two municipalities before (early 2014) and after (late 2016) expanded treatment policies were introduced.

## 2 Methods

### 2.1 Study setting and sampling

Data collection was conducted in Lekwa‐Teemane and Greater Taung municipalities within Dr. Ruth Segomotsi Mompati (RSM) District. RSM has both rural and peri‐urban areas, with approximately 230,000 total inhabitants, including approximately 93,000 adults aged 18 to 49. The estimated 503,766 PLHIV in North West, where 6.7% of the country's population resides, accounts for approximately 7.1% of the South African epidemic 13. Adult HIV prevalence in NW is currently estimated at 22.7% 5. When the research was underway, the North West had the fourth highest provincial prevalence but the lowest treatment engagement numbers in the country, with the Provincial AIDS Council estimating in 2015 that only half (51%) of PLHIV aware of their status were on ART 14. Study locations were identified in partnership with the Provincial Department of Health and represent areas with few HIV prevention and care initiatives. Surveys were conducted between January and March 2014, and again between August and November, 2016. In the interim (mid 2014–mid 2016) prevention activities were conducted in Lekwa‐Teemane, but not in Greater Taung. Activities included events offering community‐based and home‐based HIV testing, referrals to care and treatment, and screening and referrals for sexually transmitted infections and tuberculosis.

Initial sample size calculations reflected targets needed for a comparison of communities over time, with power to detect 50% increase in recent HIV testing. We employed a multistage cluster sampling approach in collaboration with Statistics South Africa to access a representative sample of municipality households. Both surveys included 23 enumeration areas (EAs) from each municipality (n = 46 EAs total) selected proportionate to size based on 2011 census data. Prior to the 2014 survey, all dwelling units (DU) in selected EAs were fully enumerated, including a detailed listing of residents. The listing was used to randomly select up to 36 inhabited dwelling units (DU) or households from each EA for inclusion (1561 DUs in total). One adult (18 to 49 years) was randomly selected per DU for participation 15. In 2016, sampled EAs were enumerated using aerial maps including all structures within the EA boundary; research teams then drove through the EAs to note structures that were no longer inhabited and/or new structures to add to the map. Within each EA, 46 DUs were randomly selected; a higher number of households were selected in 2016 to account for the probability that not all DUs would have an eligible adult. Eligibility was assessed during fieldwork: at each selected DU research staff listed all eligible residents 18 to 49 years and used a random number generator to select an individual from the household list for inclusion. Across surveys the EAs ranged in size from 14 to 355 dwellings, resulting in probability of DU selection ranging from 1.0 to 0.13. Results and detailed methods from the first survey have been published 15.

### 2.2 Data collection

For each survey, fieldworkers confirmed participant eligibility criteria (18 to 49 years, able to provide informed consent, and household residence defined as sleeping in the DU four or more nights per week); obtained written informed consent; and conducted a computer‐assisted personal interviewing (CAPI) survey at the participant's home in the preferred language (English or Setswana). The surveys elicited demographic characteristics, HIV testing and treatment history, health services utilization, sexual and other risk behaviours, and community characteristics. Participants were compensated with a mobile phone airtime voucher.

Following the survey, point‐of‐care HIV rapid antibody testing was performed by trained community health workers (CHWs) using serial HIV‐1/2 antibody rapid testing using the Alere Determine HIV‐1/2 (Alere Medical Co., Ltd, Chiba, Japan) and, if reactive, the First Response HIV 1‐2 (Premier Medical Corporation Ltd, Daman, India) 15. Participants testing positive or with discrepant results were asked to undergo lancet finger‐prick to create ten 50 μL dried blood spots (DBS) using one Munktell filter card (Ahlstrom Munktell, Helsinki, Finland) and one Whatman Protein Saver #903 card, and then referred to the nearest public health care facility. Participants who declined rapid testing were asked to provide blood for DBS for laboratory HIV diagnosis (serology: ELISA confirmed with Western blot) and offered a study number to call for the results. DBS cards were dried, stored with desiccant at ambient temperature, transported to the study laboratory within six days of collection, and stored at −70°C prior to viral load, ART exposure testing and HIV serology (when point‐of‐care antibody testing was declined or discrepant).

All procedures were approved by the Committee for Human Research at the University of California, San Francisco (UCSF), the Human Subjects Division at University of Washington; the Human Sciences Research Council Research Ethics Committee in South Africa; the Policy, Planning, Research, Monitoring and Evaluation Committee for the North West Provincial Department of Health; and the CDC's Center for Global Health, Human Research Protection.

### 2.3 Laboratory procedures

Viral load testing was performed on Munktell DBS using the COBAS AmpliPrep for sample preparation and COBAS TaqMan HIV‐1 2.0 test (Roche Applied Science, Pleasanton CA, USA; lower limit of quantification 400 copies/mL for DBS) 16. We utilize a conservative viral suppression cut off of <5000 copies/mL, which is a higher threshold than recommended for plasma due to the combined quantification of both proviral DNA (present in settings of viral RNA suppression) and cellular RNA in DBS samples 17.

ART exposure was determined using the Whatman Protein Saver #903 cards by a validated qualitative liquid chromatography MS/MS method to determine the presence of antiretroviral analytes against cutoff samples for the following three drugs: efavirenz, lopinavir, nevirapine, found in first and second‐line regimens.

### 2.4 Measures

Participant sociodemographic characteristics (sex, age, citizenship/resident status, past year employment, marital status, educational attainment and past month household food insecurity) and prior HIV testing behaviour were self‐reported. HIV status was determined through rapid or laboratory‐based HIV‐1/2 antibody testing, as described above.

Elements of the HIV care continuum were defined as follows:

*Prior knowledge of status* includes HIV‐positive participants reporting a prior positive test or ART analyte positive results. *Linked to care* was defined as reporting ever seeing a health professional for HIV care with *ideal linkage* defined as seeing a care provider and completing CD4 testing within three months of diagnosis. *Retained in care* among ART‐eligible was defined as currently on ART and seeing a care provider every three months in the past year and among ART‐ineligible was defined as seeing a care provider and undergoing CD4 testing the past year. In 2016, South Africa introduced guidance designed to encourage clinically stable patients to undergo clinical assessments only once per year and to retrieve ART for longer intervals; however, because we cannot know which patients were included in that programming, we utilize the same definition at each time point 12. *On ART* was defined as analyte‐positive for those with analyte results or by self‐reported current ART use. *Analyte‐ positive* included those DBS positive for efavirenz, lopinavir or nevirapine. *Viral suppression* was defined as DBS viral load <5000 copies/mL, although we also assess thresholds at <1000 and <3000) 17. For any participants with conflicting information (e.g. reported unknown status but tested positive for ART analytes), we prioritized the objective marker over self‐report, as individuals may either not have understood the questions or chosen not to disclose for their own reasons 18.

### 2.5 Analysis

All analyses accommodated the multi‐stage survey design, including clustering and weighting 19. Weights were created using the inverse probability of selection at each stage (EA, DU and person) and adjusted for non‐response to reflect municipality age and sex distributions 20. We calculated proportions and 95% confidence intervals (CIs) to describe overall and sex‐specific demographic characteristics, HIV testing, prevalence and care engagement. One study participant self‐reported as transgender in 2016; because sampling was based on biological sex, this individual was included in subsequent analyses in their biological sex group (male). We estimated chi‐square statistics using the second‐order Rao and Scott correction for bivariate analyses 21. Given the aim to evaluate a comprehensive prevention programme, we first estimated difference‐in‐differences models to assess changes over time within and by municipalities using generalized linear mixed effects logistic regression models, including a random intercept for enumeration area, our stratification variable and the Huber variance estimator for cluster‐robust standard error estimation 22. We found that reported past‐year HIV testing was statistically higher in Lekwa‐Teemane as compared to Greater Taung; however, care continuum outcomes, including known HIV status, did not change differentially by municipality. We therefore present combined municipal data in the current analysis comparing 2014 to 2016 data. We used generalized linear regression modelling (GLM) with log binomial models to evaluate differences in the HIV care continuum between 2014 and 2016. We first imputed missing data on HIV status and DBS outcomes (analyte and viral load) using multiple imputation by chained equations procedures, employing 50 datasets from models incorporating socio‐demographic, clinical and behavioural predictors. To account for our study design, imputation models included enumeration area and survey weights, and main analyses employed survey procedures 23. Differences where *p *<* *0.05 were considered statistically significant. Analyses were performed using Stata version 14 (StataCorp, College Station, TX, USA).

We conducted sensitivity analyses to ensure that any differences in sample composition between the two surveys did not influence findings. Using inverse probability weights, we reweighted the 2014 data to make it comparable to the 2016 sample in terms of the following participant characteristics: age group, educational attainment, marital status, South African citizenship, past year income and past month food insecurity. Final stabilized weights also accounted for the sex and age‐specific probability of being in one's observed survey year, whether 2014 or 2016. This approach is mathematically equivalent to direct standardization (i.e. by age) to encompass multiple differences in underlying demographics 24, 25.

### 2.6 Role of the funding source

This project has been supported by the U.S. President's Emergency Plan for AIDS Relief (PEPFAR) through the U.S. Centers for Disease Control and Prevention (CDC) under the terms of Cooperative Agreement 5U2GGH000324. Additional support was received through the UCSF‐Gladstone Institute of Virology & Immunology Center for AIDS Research (CFAR), an NIH‐funded programme (P30 AI027763). The CDC reviewed the protocol. Funders had no involvement in the data collection, interpretation, writing or the decision to submit a manuscript.

## 3 Results

In 2014, 43 of the 46 selected EAs were successfully enumerated; fieldworkers were not granted access to three farm areas. Of the 1527 enumerated DUs, 98.5% were approached; contact was made at 91.7%, yielding 1146 eligible individuals. A total of 1048 (91.0% of eligible participants) consented to participate; four were later determined ineligible (due to fieldworker error enrolling a non‐selected DU member), resulting in a total sample of 1044. Median cluster size was 25 (IQR: 22 to 28). In 2016, we approached 1925 DUs (95.7% of those targeted) in 46 selected EAs and were able to assess 1571 (81.6% of those approached) for study eligibility; 1060 (67.5%) dwellings had at least one eligible household member. A total of 980 participants (92.5%) enrolled; six were later determined ineligible (enrolment of non‐selected DU or DU member), data were lost for two surveys due to errors saving and uploading the survey files, and one survey was not matched to a household, resulting in an analytic sample of 971 participants. Median cluster size in 2016 was 21.5 (IQR: 17 to 27). Most common reasons for declining participation in both surveys were reported disinterest, lack of time and not feeling well. Across the surveys, 77.1% consented to HIV testing through rapid test or DBS (n = 1555).

Just over half of study participants were female (53.0%) and 49% were aged 18 to 29 years. (Table 1). Nearly all participants were South African citizens or permanent residents. The only characteristic that differed significantly between 2014 and 2016 samples was past year employment, at 54% and 30% in 2014 and 2016 respectively. Most participants were single or non‐cohabitating (70.3% and 72.5% in 2014 and 2016 respectively), and one quarter reported past month food insecurity (25.7% and 23.0% respectively).

**Table 1:** Socio‐demographic characteristics of study participants in 2014 and 2016, North West Province, South Africa

	2014 Survey			2016 Survey			*p* value
	n = 1044			n = 971			
	n	weighted %	95% CI	n	weighted %	95% CI	
Sex							
Male	401	47.0	41.7–52.2	393	46.8	42.0–51.7	1.000
Female	643	53.0	47.8–58.3	577	53.0	48.1–57.9	
Trans	0	‐	‐	1	0.1	0.0–1.0	
Age group							
18 to 29 years	453	49.0	45.2–52.8	514	49.0	44.5–53.4	1.000
30 to 39 years	344	28.0	24.9–31.2	289	28.1	24.1–32.4	
40 to 49 years	247	23.0	20.0–26.0	168	23.0	18.3–28.4	
South African citizen or permanent resident^a^	1035	99.4	98.9–99.9	966	99.6	98.4–99.9	0.682
Municipality							
Greater Taung	557	73.8	63.6–82.0	527	73.8	71.9–83.1	1.000
Lekwa‐Teemane	487	26.2	18.0–36.4	444	26.2	16.9–38.1	
Employed past 12 months	629	53.9	48.8–59.0	289	30.0	25.5–35.0	<0.001
Marital status							
Married/living with partner	321	25.8	21.2–30.4	243	23.5	18.7–27.7	0.792
Single/in relationship	675	70.3	65.7–75.0	688	72.5	68.1–76.5	
Single (separated/divorced)	27	2.1	1.1–3.1	24	2.4	1.5–2.7	
Single (widowed)	21	1.7	0.9–2.5	16	1.7	0.9–3.1	
Educational attainment							
Primary or less	241	21.0	16.8–25.2	178	18.7	14.7–23.6	0.288
Some secondary	431	43.7	38.5–48.9	417	41.6	36.9–46.6	
Completed secondary	299	27.6	23.2–32.1	322	34.2	29.8–38.9	
College/university or technikon	73	7.7	3.4–12.1	54	5.4	3.2–9.0	
Any food insecurity past month	258	25.7	20.3–31.0	205	23.0	17.3–29.9	0.520

Weights account for sampling, non‐response and age/sex of target population, including size of municipality.

a Missing responses for three participants.

### 3.1 HIV prevalence and testing behaviours

HIV prevalence did not vary significantly across time at 22.8% (95% CI 18.2–27.5) in 2014 and 21.2% (CI 16.7–25.6) in 2016 (Table 2). HIV prevalence was consistently lower for men compared to women at 18.7% (CI 12.7–24.7) versus 26.5% (CI 21.3–30.9; *p *=* *0.029) in 2014 and 15.0% (CI 9.5–20.5) versus 26.6% (CI 20.4–32.8; *p *=* *0.006) respectively. At both time points, approximately four‐fifths of study participants reported having ever tested for HIV (79.7% and 81.3% respectively; Table 2), with higher proportions of females reporting ever testing. Data on past‐year HIV testing suggested an increasing but not significant trend over time; overall, 51.0% (CI 46.4–55.6) of 2014 participants and 55.0% (CI 50.3–59.5) of 2016 participants reported past‐year HIV testing; men consistently reported less testing than women.

**Table 2:** Engagement in the HIV testing and care continuum, 2014 to 2016, overall and by gender in 2014 and 2016, North West Province, South Africa

	Overall			Male			Female		
	2014	2016	*p* value	2014	2016	*p* value	2014	2016	*p* value
	weighted % (95% CI)	weighted % (95% CI)		weighted % (95% CI)	weighted % (95% CI)		weighted % (95% CI)	weighted % (95% CI)	
All participants									
HIV Prevalence^a^^,^ b	22.8 (18.2–27.5)	21.2 (16.7–25.6)	0.613	18.7 (12.7–24.7)	15.0 (9.5–20.5)	0.387	26.5 (21.3–30.9)	26.6 (20.4–32.8)	0.986
HIV tested ever	79.7 (75.1–83.6)	81.3 (75.9–85.7)	0.627	68.9 (62.0–75.0)	68.8 (60.1–76.4)	0.993	89.2 (84.4–92.6)	92.3 (88.1–95.1)	0.244
HIV tested past 12 months^c^	51.0 (46.4–55.6)	55.0 (50.3–59.5)	0.093	42.0 (35.8–48.5)	45.1 (38.1–52.3)	0.444	62.3 (56.0–68.2)	70.2 (63.5–76.2)	0.079
HIV positive participants^d^									
Known status	68.7 (60.9–75.6)	72.8 (63.6–80.4)	0.474	53.8 (40.0–67.0)	56.0 (41.0–70.0)	0.830	77.3 (64.7–86.3)	81.1 (68.9–89.3)	0.614
Linked to care									
Minimal^e^	66.5 (58.6–73.6)	71.6 (62.6–79.1)	0.374	49.3 (35.5–63.3)	54.7 (40.0–68.7)	0.604	76.4 (64.0–85.4)	79.9 (67.6–88.3)	0.641
Ideal^f^	35.6 (27.2–45.0)	21.9 (14.9–31.0)	0.034	27.8 (17.6–41.0)	13.8 (4.7–34.3)	0.210	40.1 (30.4–50.5)	25.9 (16.4–38.3)	0.086
Retained in care^g^	49.2 (42.2–56.3)	58.4 (48.1–68.0)	0.139	33.1 (21.7–46.9)	44.9 (32.3–58.4)	0.220	58.4 (49.4–66.9)	65.0 (51.8–76.3)	0.389
On ART	56.2 (48.7–63.3)	67.2 (57.9–75.4)	0.056	43.5 (31.5–56.2)	50.2 (37.0–63.4)	0.472	63.4 (52.2–73.4)	75.6 (63.4–84.7)	0.117
Self‐reported	52.0 (44.3–59.5)	59.7 (59.6–69.0)	0.215	35.3 (24.1–48.4)	44.9 (32.2–58.4)	0.305	61.5 (50.5–71.4)	66.9 (54.3–77.5)	0.494
ART analyte positive^b^	51.0 (43.5–58.5)	62.6 (52.9–72.2)	0.058	33.4 (21.6–45.3)	45.9 (32.4–59.3)	0.178	61.1 (50.2–72.0)	70.8 (58.7–83.0)	0.234
Virally suppressed									
<5000 copies/mL^b^	37.3 (28.4–46.2)	63.3 (54.6–72.0)	<0.001	17.3 (6.8–27.7)	45.8 (27.0–64.7)	0.010	48.8 (38.3–59.2)	72.0 (63.8–80.1)	0.002
<3000 copies/mL^b^	30.3 (21.6–39.0)	58.3 (49.2–67.4)	<0.001	12.2 (2.3–22.2)	40.6 (24.4–56.8)	0.010	40.6 (30.2–51.0)	67.1 (58.0–76.1)	0.001
<1000 copies/mL^b^	16.6 (9.3–23.8)	41.7 (33.6–49.8)	<0.001	7.8 (0.7–14.9)	28.0 (13.3–42.7)	0.017	21.6 (11.7–31.5)	48.4 (39.0–57.9)	0.002

Weights account for sampling, non‐response and age/sex of target population.

^a^Includes self‐report and confirmed; ^b^missing data multiply imputed; ^c^does not include those who were diagnosed HIV positive prior to 12 months in the denominator; ^d^Denominators include the full HIV‐positive population; ^e^minimal linkage to care defined as ever having seen a health care provider about HIV; ^f^ideal linkage to care defined as having seen a health care provider about HIV and receiving CD4 testing, within the three months following HIV diagnosis; ^g^retained in care for pre‐ART phase defined as seeing a provider and obtaining CD4 testing at least once per year; retained in care for the ART phase defined as seeing a provider at least every three months and being on ART.

### 3.2 The HIV care continuum

We assessed changes to the HIV care continuum over time among the HIV‐positive population to assess improvements in engagement in care (Table 2). Both the proportion of HIV‐positive participants with known status and the proportion linked to care were similar over time, although reported linkage within three months of diagnosis fell among both men and women (Table 2). Retention in care increased from 49.2% in 2014 to 58.4% in 2016, but was not statistically significant. Similarly, the proportion either reporting being on ART or with evidence of ART analytes increased from 56.2% to 67.2%; this difference was marginally statistically significant (*p *=* *0.056). Increases observed over time in the proportion of PLHIV who tested analyte positive, indicating ART uptake and use, were also marginally significant at 51.0% (CI 43.5–58.5) in 2014 compared to 62.6% (CI 52.9–72.2) in 2016 (*p *=* *0.058). Large improvements in viral suppression across time were observed. In 2014, 37.3% (CI 28.4–46.2) of all PLHIV were virally suppressed at <5000 copies/mL compared to 63.3% (CI 54.6–72.0) in 2016 (*p *<* *0.001); with similar improvements in suppression across thresholds and large improvements for both sexes. The HIV care continuum is also presented by sex in Figures S1 and S2.

### 3.3 90‐90‐90 targets

**Figure 1:**
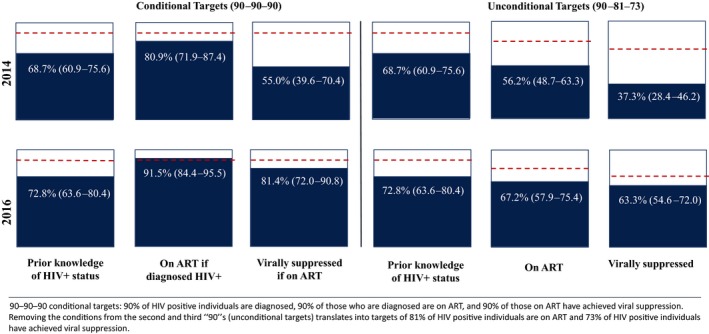
Progress towards 90‐90‐90 Targets: conditional and unconditional (Composite) knowledge of HIV status, ART initiation and viral suppression in 2014 and 2016, North West Province, South Africa.

Prior knowledge of HIV status among all HIV positive individuals (the first “90”) was consistent over time at 68.7% (CI 60.9–75.6) in 2014 compared to 72.8% (CI 63.6–80.4) in 2016 (Table 3; Figure 1). Prior knowledge of status was consistently lower among men both in 2014 and 2016. The proportion of PLHIV who knew their status that had evidence of currently being on ART (the second “90”) increased significantly from 80.9% (CI 71.9–87.4) in 2014 to 91.5% (CI 84.4–95.5) in 2016. Increases were marginally statistically significant for women (*p *=* *0.060) and improvements were not significant for men (*p *=* *0.287). Among those individuals on ART, viral suppression (the third “90”) increased significantly over time; 55.0% (CI 39.6–70.4) of the sample had viral load <5000 copies/mL in 2014 compared to 81.4% (CI 72.0–90.8) in 2016. There was a particularly large increase observed for males (33.7% to 81.6%), bringing viral suppression to equivalent levels among men and women by 2016. Despite the improvements in men engaging in care, overall differences in the achievement of 90‐90‐90 targets by sex are largely due to the stark disparities in testing and knowledge of HIV status, with HIV‐positive men less likely to have been diagnosed. This is evidenced in the composite target of having 73% of all PLHIV virally suppressed. Using our imputation model (instead of multiplying out the three 90s), our findings indicate that 63.3% of PLHIV in the research area overall were virally suppressed; however, 72.0% of women achieved viral suppression – very close to the 73% target and far outperforming men at 45.8% (represented in Figure 1).

**Table 3:** 90‐90‐90 indicators: knowledge of HIV status, ART initiation, and viral suppression overall and by gender in 2014 and 2016, North West Province, South Africa

	Overall			Male			Female		
	2014	2016	*p* value	2014	2016	*p* value	2014	2016	*p* value
	weighted % (95% CI)	weighted % (95% CI)		weighted % (95% CI)	weighted % (95% CI)		weighted %	weighted %	
HIV‐positive participants									
Prior knowledge of HIV‐positive status	68.7 (60.9–75.6)	72.8 (63.6–80.4)	0.474	53.8 (40.0–67.0)	56.0 (41.0–70.0)	0.830	77.3 (64.7–86.3)	81.1 (68.9–89.3)	0.614
On ART^a^	80.9 (71.9–87.4)	91.5 (84.4–95.5)	0.031	78.7 (59.2–90.4)	88.6 (77.8–94.5)	0.287	81.7 (70.6–89.2)	92.4 (83.7–96.7)	0.060
Viral suppression (<5000)^b^^,^ c	55.0 (39.6–70.4)	81.4 (72.0–90.8)	0.012	33.7 (11.4–56.0)	81.6 (59.4–100.0)	0.016	62.5 (47.2–77.8)	81.3 (72.1–90.6)	0.056
<3000 copies/mL^b^^,^ c	42.6 (28.0–57.1)	74.3 (62.3–86.2)	0.005	20.6 (0.8–40.4)	70.8 (49.2–92.5)	0.017	50.3 (34.2–66.4)	75.4 (61.7–89.1)	0.032
<1000 copies/mL^b^^,^ c	28.1 (16.0–40.3)	52.3 (40.5–64.1)	0.014	19.5 (1.5–37.6)	49.4 (22.7–76.0)	0.086	31.2 (17.2–45.1)	53.3 (40.5–66.1)	0.042

Weights account for sampling, non‐response and age/sex of target population.

^a^Among those with prior knowledge of HIV status; ^b^among those on ART; includes those self‐reporting ART and those who tested positive for ART analytes; ^c^missing data multiply imputed.

Sensitivity analyses standardizing the 2014 to the 2016 population did not substantively change our results, with the exception of recent HIV testing: the difference in the proportion reporting past‐year HIV testing in sensitivity analysis was 54.8% versus 61.4% (*p *=* *0.025) in 2014 versus 2016 as compared to 51.0% versus 55.0% (*p *=* *0.093) in the main analysis. However, findings regarding the care cascade and 90‐90‐90 targets remained consistent in sensitivity analyses, including the absence of differences in known status over time, implying that while there was potentially more testing underway in 2016 as compared to 2014, this did not translate into more diagnoses.

## 4 Discussion

Using data from recent consecutive population‐representative surveys in two municipalities in North West Province, South Africa, we found strong evidence that HIV care engagement has improved in recent years. We found notable advances in both the second and third 90‐90‐90 targets. The proportion of PLHIV with known status who are on treatment reached 91% and the proportion of virally suppressed on treatment is estimated at 81%. Although our results suggest that prevention activities led to a higher proportion of individuals reporting HIV testing, these activities did not result in improvements in the first 90 target. Increased testing alone did not improve the proportion of PLHIV who knew their status, which remained similar over time at 69% in 2014 and 73% in 2016. Our findings suggest that shifts in the national programme are the drivers of change in these communities, as opposed to introduction of local activities designed to enhance testing and engagement in care. This finding is consistent with recent community trials, such as the ANRS Treatment as Prevention (TasP) trial in South Africa, which similarly found no difference in ART coverage in communities receiving a test and treat intervention as compared to a control condition, but demonstrated improvements overall between 2012 and 2016 during treatment expansion 26.

Our estimates of known status, the first 90, remained consistent at around 55% of men and 80% of women in 2014 and 2016, with only small, non‐significant increases. Our estimates are slightly higher than those from a comparable survey in KwaZulu‐Natal, where 52% of HIV‐positive men and 65% of women knew their status in 2014 to 2015 27, lower than another, similar study noting 68% of status knowledge among men, 28 and much lower than the 91.5% (overall) reported in the ANRS TasP trial 26. Our estimates are slightly lower than 2015 to 2016 national estimates of 74% with known status 29, and much lower than modelled estimates from mid‐2016 of 84% to 89% across provinces 30. They also diverge considerably from the 2017 National HIV Survey Summary, which estimated that 78.0% and 88.9% of HIV‐positive males and females respectively are aware of their status 5. The first “90” is the most difficult proportion to estimate as high quality data on the number of people who are living with HIV (the total population who should be included in the denominator), are not available 31. Also difficult to come by are precise estimates of how many diagnoses have been made (those who should also be in the numerator). Other discrepancies and the wide range of estimates of the first 90 might be due to large regional differences in testing uptake; although national and regional data in this case are similar. Nonetheless, our findings contribute to growing evidence that diagnosis remains a major barrier to achieving 90‐90‐90 32 and to the increasing consensus that given the large disparity in knowledge of status among men, who test less frequently and who have poorer HIV outcomes over the lifecourse as compared to women 33; new programming must creatively target men 9, 34, 35.

Our findings regarding the second 90 target, “on ART,” that 88.6% of men and 92.4% of women with known status are on treatment, are higher than similar surveys 27, 28 and national estimates 5. National HIV Survey Summary data estimate only 67.4% and 72.2% of males and females, respectively are on ART. However, this discrepancy is likely due to differences in calculating the denominator; if more people are believed to know their status, then the number on treatment will reflect a smaller proportion. Conversely, if fewer people are believed to know their status, the estimate of the proportion on treatment will be higher. Estimates of the third 90 in South Africa are reliably over 80% 5, 26, 27, which is consistent with our data. Our findings on the overall proportion of PLHIV who are virally suppressed, with 46% of men and 72% of woman living with HIV reaching viral suppression, are higher than the national survey findings of 43% and 58% of male and female PLHIV achieving suppression 5, and higher than the modelled provincial data for North West Province 36. These differences may be due to our use of an unconditional imputation model, as opposed to simple multiplication of the 90‐90‐90 estimates. Our model allows for HIV‐positive participants who may not know their status or be on treatment to have low viral loads suggestive of suppression, as this was observed in our data and has been found elsewhere 37. Additionally, our higher estimate of total viral suppression could be due to utilizing the higher viral suppression threshold of < 5000, based on the sample medium.

These data were collected during rapid treatment scale up and multiple treatment guideline revisions. The South African National Department of Health (NDoH) introduced the first line FDC, single pill (TDF/FTC/EFV), in 2013. As the new FDC was implemented in stages, it is likely that some patients in rural areas were still being transitioned to FDC in early 2014, at the time of the first survey, whereas by 2016 all clients would have been on a FDC regimen. Single‐pill regimens have been found to improve medication adherence 38, increase patient satisfaction 39, and can improve clinic attendance 40. Medication monitoring and storage is easier when patients and providers need to keep track of only one pill. Furthermore, the NDoH extended ART qualifying criteria twice in the interim of the two surveys, with implementation of treatment access for those with CD4 <500 in 2015, after the first survey, and universal access to care implemented approximately as the second survey began. While it is unlikely that universal access was implemented uniformly and immediately with the announcement, the ART expansion in 2015 likely had a large impact on increased the number of people on treatment and suppressed between the surveys. During this time, the government also introduced new adherence guidelines with efforts to streamline treatment, providing means to more rapid and longer ART prescribing for stable patients, along with introduction of strategies such as community adherence clubs in 2016. Finally, the health department also published more extensive guidance around tracing patients defaulting from care, including a push for PEPFAR partners to oversee implementation of partner tracing, which might have contributed to re‐engagement of patients.

In the full HIV‐positive population, we noted an approximately 9% point increase in reported retention in care (*p *=* *0.139), a 12% point increase in the presence of ART analytes (*p *=* *0.058), and 26% point increase in viral suppression (*p *<* *0.001). We believe that the structural, policy‐level changes are largely responsible for the improvement in ART adherence and viral suppression. Others have attributed similar findings on improvements in initiation rates and therapeutic response to changes in guidelines 32. It is also possible that improvements in the later “90”s may be due to changes in social context of HIV treatment, including reduced stigma or increased comfort with disclosure and support seeking.

This study compared two sequential, population‐representative samples of the general population aged 18 to 49 years in North West Province, South Africa during a period of rapid policy transition. Our findings are representative of an area of comprising 230,000 people and provide insight into the impacts of policy changes in South Africa's HIV response in a district far from the major metropolitan areas. Our findings are subject to some biases. While fewer than 10% of eligible participants declined survey participation, 23% declined HIV testing in our survey. Our participation rates are still higher than the national survey, in which only 67.7% and 58.4% of men and women respectively agreed to HIV testing 5. We accounted for non‐response and multiply imputed missing data; however, these estimates are subject to bias if refusals were differential by HIV‐status 41. Self‐reported knowledge of status, linkage to care and retention in care is subject to misreporting. Guidance around the frequency of visits changed for stable patients between the surveys, which likely explains why our estimate of retention is lower than our estimate of those positive for ART analytes in 2016. Viral suppression is estimated from DBS; we used a high (>5000 copies/mL) threshold for defining viral suppression, as cellular proviral HIV DNA and virion RNA both contribute to copy number when using DBS instead of plasma from whole blood 17. As a result, viral suppression estimates might be inflated. However, we see similar improvements in viral suppression using all thresholds. Furthermore, while there were some differences between 2014 and 2016 samples, sensitivity analysis aimed at improving sample comparability yielded similar results.

## 5 Conclusions

Over the span of two and a half years during which the first line FDC was introduced, CD4 thresholds were steadily removed for ART eligibility, and guidance was published on facilitating treatment and tracking defaulting patients, we found that the second 90‐90‐90 target was reached and the third is within reach in a rural area of North West Province, South Africa. We conclude that South Africa's policies have improved and facilitated access to treatment. No significant improvements were evident in the first 90 target, despite testing campaigns in the area and increases in reported HIV testing. Our findings indicate that by late 2016, women in the research area had almost achieved the 73% suppression target. Our data also indicate that woman far outperform men in meeting this target, a finding common to recent large‐scale trials 42. It is possible that South Africa can meet the ambitious targets, but only if detection of infection among men is improved. This will require targeted and differentiated testing models to encourage uptake of HIV testing by men and others less inclined to attend a clinic. Accordingly, South Africa's new National Strategic Plan 43 calls for expanded testing services delivered outside health facilities, including self‐screening 44, with efforts to target men, adolescents and other populations not accessing testing services to close testing gaps. By expanding testing models beyond current clinic‐based approaches and pairing testing with facilitated linkage strategies, South Africa may be able in a position to meet its targets and grow closer to ending the threat of the HIV epidemic.

## Competing interests

The authors declare no conflicts of interest.

## Authors’ contributions

SAL, SBS, JSG, AP, TL, EV and SB conceived of this study. SAL, JSG, MJR, JLM and EN were responsible for study implementation and supervision. AP and TL were responsible for laboratory protocols and quality control. AME and EA were responsible for data merging and management. AME led the analysis with assistance from EA, SAL and SS. Data were interpreted by SAL, AME, JSG, AP, TL, WDFV, EN, SB and SS. SAL and AME wrote the paper. All authors reviewed, edited and approved the final manuscript.

## Supporting information


**Figure S1.** HIV Care Continuum among all HIV‐positive Males in 2014 and 2016, North West Province, South Africa.
**Figure S2.** HIV Care Continuum among all HIV‐positive Females in 2014 and 2016, North West Province, South Africa.
