# Supplementary material for: Improvements in the South African HIV care cascade: findings on 90‐90‐90 targets from successive population‐representative surveys in North West Province
Source: J Int AIDS Soc. 2019 Jun 12;22(6):e25295. doi: 10.1002/jia2.25295 (PMC6562149; doi:10.1002/jia2.25295)
Supplement: Supplementary file 1 — Figure S1. HIV Care Continuum among all HIV‐positive Males in 2014 and 2016, North West Province, South Africa. Figure S2. HIV Care Continuum among all HIV‐positive Females in 2014 and 2016, North West Province, South Africa. [file JIA2-22-e25295-s001.docx]

**Supplemental File: Figure 2. HIV Care Continuum among all HIV-positive Males in 2014 and 2016, North West Province, South Africa.**


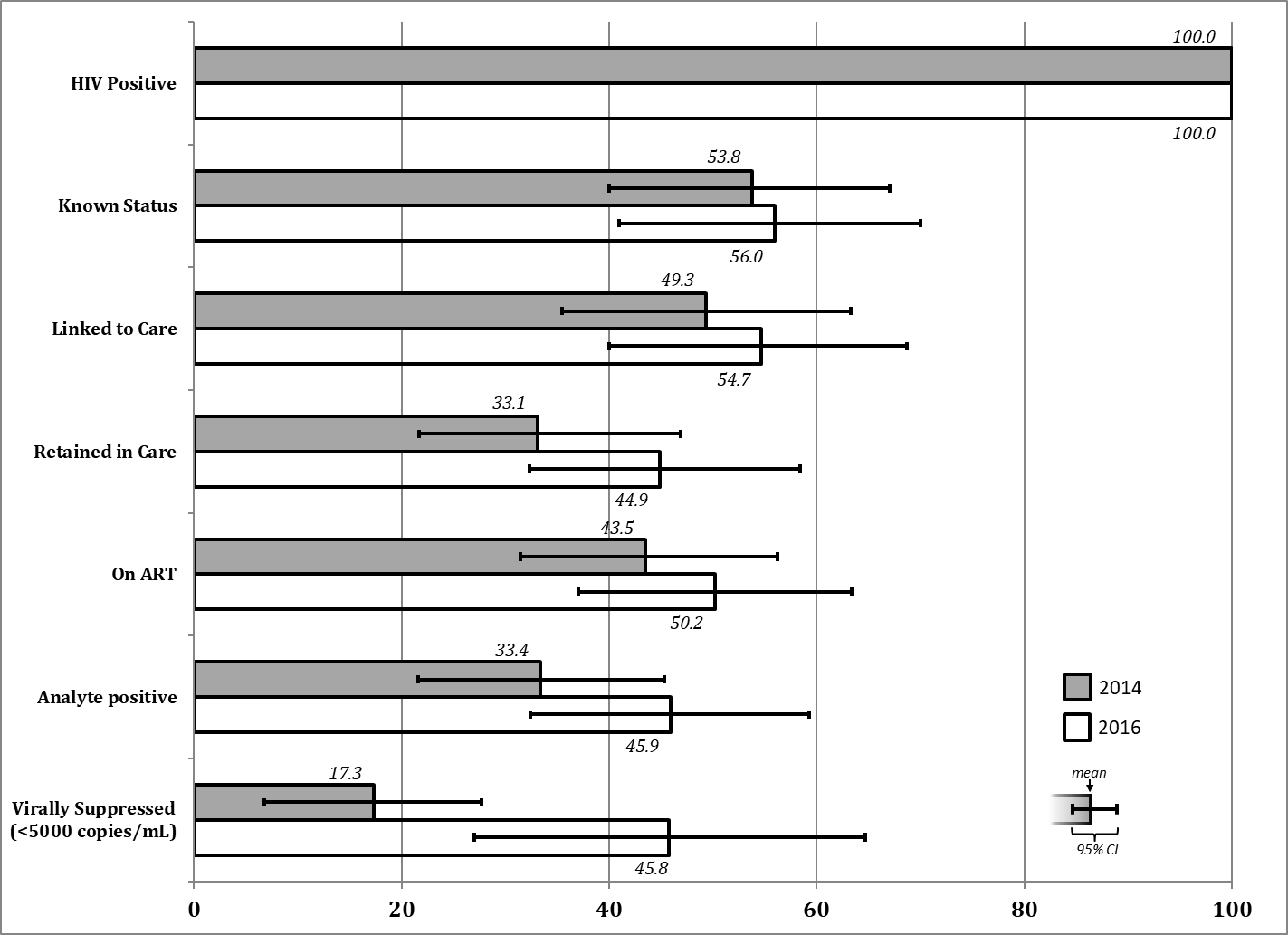


**Supplemental File: Figure 3. HIV Care Continuum among all HIV-positive Females in 2014 and 2016, North West Province, South Africa.**

**
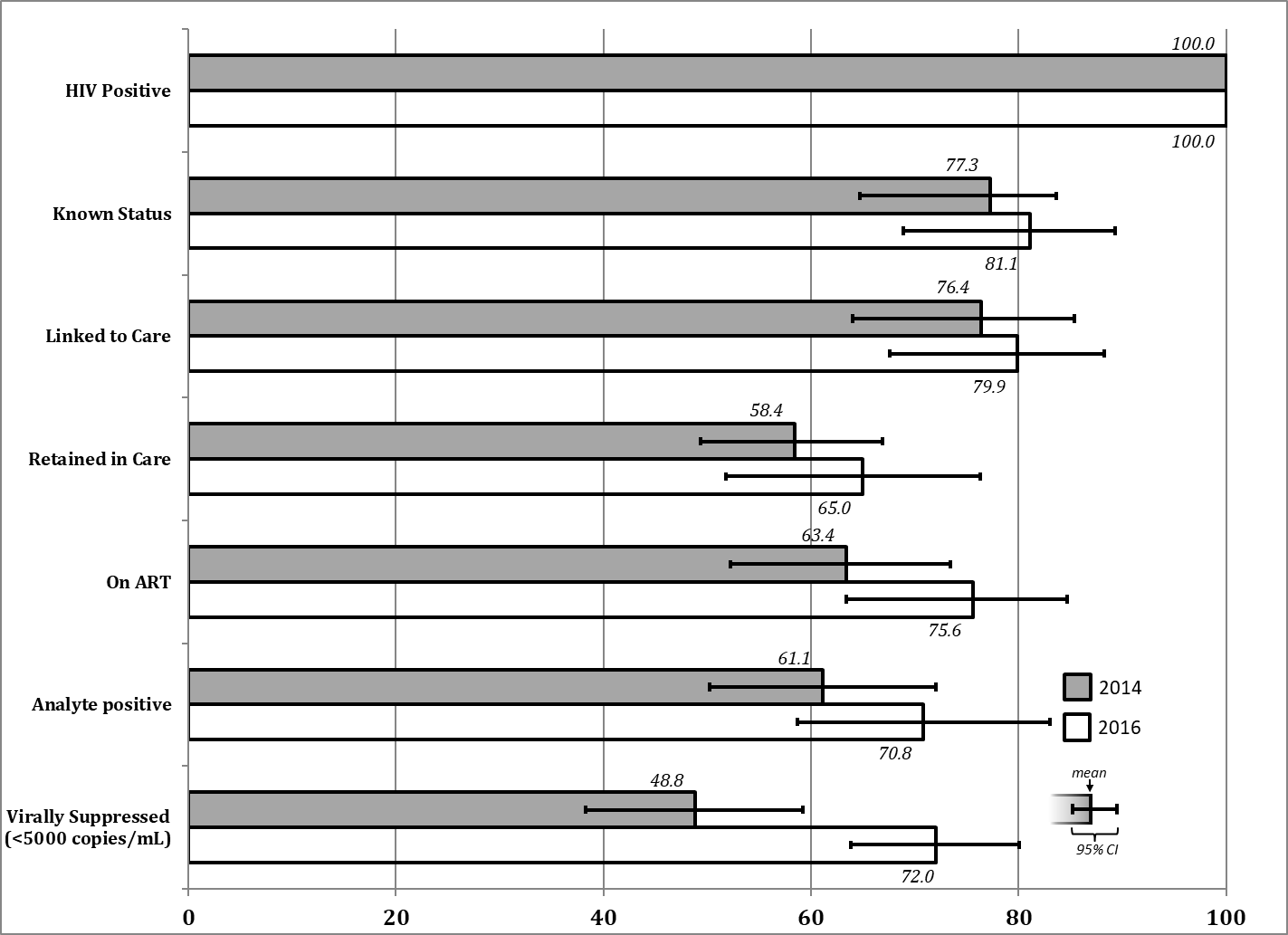
**
